# Air pollution and venous thrombosis: a meta-analysis

**DOI:** 10.1038/srep32794

**Published:** 2016-09-07

**Authors:** Liang Tang, Qing-Yun Wang, Zhi-Peng Cheng, Bei Hu, Jing-Di Liu, Yu Hu

**Affiliations:** 1 Institute of Hematology, Union Hospital, Tongji Medical College, Huazhong University of Science and Technology, Wuhan, Hubei, 430022, China; 2 Collaborative Innovation Center of Hematology, Union Hospital, Huazhong University of Science and Technology, Wuhan, 430022, China

## Abstract

Exposure to air pollution has been linked to cardiovascular and respiratory disorders. However, the effect of air pollution on venous thrombotic disorders is uncertain. We performed a meta-analysis to assess the association between air pollution and venous thrombosis. PubMed, Embase, EBM Reviews, Healthstar, Global Health, Nursing Database, and Web of Science were searched for citations on air pollutants (carbon monoxide, sulfur dioxide, nitrogen dioxide, ozone, and particulate matters) and venous thrombosis. Using a random-effects model, overall risk estimates were derived for each increment of 10 μg/m^3^ of pollutant concentration. Of the 485 in-depth reviewed studies, 8 citations, involving approximately 700,000 events, fulfilled the inclusion criteria. All the main air pollutants analyzed were not associated with an increased risk of venous thrombosis (OR = 1.005, 95% CI = 0.998–1.012 for PM_2.5_; OR = 0.995, 95% CI = 0.984–1.007 for PM_10_; OR = 1.006, 95% CI = 0.994–1.019 for NO_2_). Based on exposure period and thrombosis location, additional subgroup analyses provided results comparable with those of the overall analyses. There was no evidence of publication bias. Therefore, this meta analysis does not suggest the possible role of air pollution as risk factor for venous thrombosis in general population.

Global air pollution is a leading problem for public health^1^,^2^. It is caused by a number of environmental air pollutants including carbon monoxide (CO), nitrogen dioxide (NO_2_), sulfur dioxide (SO_2_), ozone (O_3_), and particulate matter (PM)^3^. Common constituents of PM are nitrates, sulfates, elemental and organic carbon, organic compounds, endotoxin, cell fragments, and a variety of metals. Ambient PM is distinguished, according to aerodynamic diameter, in coarse (PM_10_, ≤10 μm) and fine (PM_2.5_, ≤2.5 μm) particles. The potentially deleterious effects of air pollution on cardiovascular and respiratory health have been suspected for more than half a century. Over the past two decades, mounting epidemiological and mechanistic studies have provided convincing evidence that both acute and chronic exposure to main air pollution, especially by particulates, increases the risk of cardiovascular morbidity and mortality^4^,^5^,^6^. The World Health Organization reported that ambient air pollution contributed to 1.3 million deaths in the world in 2008^7^. Moreover, the Global Burden of Disease Study showed that air pollution was responsible for more than 1.2 million premature deaths in China in 2010^8^.

Annually, venous thrombosis occurs in approximately 1:1000 people in European countries^9^,^10^. Thrombosis arises most frequently as deep vein thrombosis and pulmonary embolism. Several genetic risk factors have been identified to cause a lifelong hypercoagulable state among people with ethnicities^11^,^12^,^13^,^14^,^15^. Thromboembolism tends to occur when one or more of the acquired conditions come into play^16^,^17^,^18^. Is air pollution one such acquired risk factor for venous thrombosis? Although air pollution is linked to an elevated risk of cardiovascular diseases, such as myocardial infarction and heart failure, the association between air pollution and risk of venous thrombosis remains controversial^19^.

Therefore, the aim of this study is to investigate the associations between main air pollutants and risk of venous thrombosis. We systematic reviewed the available literature and performed a meta analysis with Stata 11.0 software, according to MOOSE guidelines^20^,^21^.

## Methods

This study is registered in PROSPERO (http://www.crd.york.ac.uk/PROSPERO/) with register number of CRD42014015301.

### Databases.

We searched Pubmed, Embase, EBM Reviews, Healthstar, Global Health, Nursing Database, and Web of Science using the terms related to the type of exposure (air pollution, ozone, carbon monoxide, nitrogen dioxide, sulfur dioxide, PM_10_, and PM_2.5_) and to the type of outcome (venous thrombosis, venous thromboembolism, deep vein thrombosis, pulmonary embolism, and pulmonary thromboembolism). The full search criteria are provided in the Supplemental Material. Furthermore, we supplemented citations by cross checking the reference lists of eligible studies and relevant reviews to identify additional published and unpublished data.

### Study selection and data extraction

All studies in human beings that presented original data and were published in full text, or meeting abstract were eligible for inclusion, with no restrictions on publication date, language, or ethnicity. We excluded animal studies, *ex vivo* and toxicological studies, commentaries and editorials, case reports, and studies with no original data. If a citation is lacking of enough quantitative data and these essential data could not be obtained from the correspondent author, the study was excluded. Two investigators (L.T. and Q.Y.W.) screened all citations for potentially eligible studies and extracted data independently. Disagreements were adjudicated by a third investigator (B.H.).

### Study quality assessment

The Newcastle-Ottawa Scale, with some modifications was adapted to judge study quality, according to validated scales in previous studies and the Cochrane Collaboration^22^,^23^. We evaluated 10 items as follows: (1) VT diagnosis: We considered the diagnosis to be validated if it was coded according to the International Classification of Diseases or based on objective investigations (color Doppler ultrasonography or vein angiography for deep vein thrombosis, CT angiography or ventilation/perfusion lung scan for pulmonary embolism). For cases reported in registries, we considered the diagnosis as validated. (2) Pollutant measurement: Good quality was considered if measurement was performed at least daily. (3) Study area: Complete evaluation of study area was referred to as a study where the unit of analysis for the exposure matched that of the outcome. (4) Total population: Good quality was considered if the study population was not limited to some special groups. (5) Multiple lags: Multiple lags were defined as studies that evaluated pollutant levels in a distributed lag model beyond lag of 1 day (in short-term analyses) or 1 year (in long-term analyses). (6) Repeated events: Good quality was considered if repeated events were controlled. (7)–(9) Temperature, Time trends, and Season: Good quality was considered if adjustment has been made. (10) Other factors: Good quality was considered if additional confounding factors were adjusted, such as humidity, day of week, smoking, body-mass index, and cancer. For each item, 1 was given to good quality, while 0 was given to low quality.

### Data synthesis

Adjusted odds ratios (OR) for venous thrombosis were pooled for a standardized increment in pollutant concentration of 10 μg/m^3^. This level is the one that is used most frequently. If the increment in pollutant concentration was not 10 μg/m^3^, standardized risk estimates were calculated using the following formula: OR_(standardized)_ = OR^Increment (10)/Increment (original)^. Most studies have verified a linear relation between air pollutants increases and venous thrombosis risk. Due to the significant heterogeneity between studies, we estimated RRs and 95% CIs using a random-effects model. To further assess heterogeneity, subgroup analyses were performed. Statistical heterogeneity across the studies was examined using the standard *I*^*2*^statistic and Q-test. Because this test has limited power when the number of studies is small, the presence of heterogeneity was considered at a significance level of 0.10. Publication bias was assessed using Egger’s regression test and Begg’s test^24^. All tests were 2-sided and statistical significance was defined as *P* ≤ 0.05. Analyses were performed with Stata SE/MP 11.0 (StataCORP, College Station, TX, USA).

## Results

### Included studies

**Figure 1:**
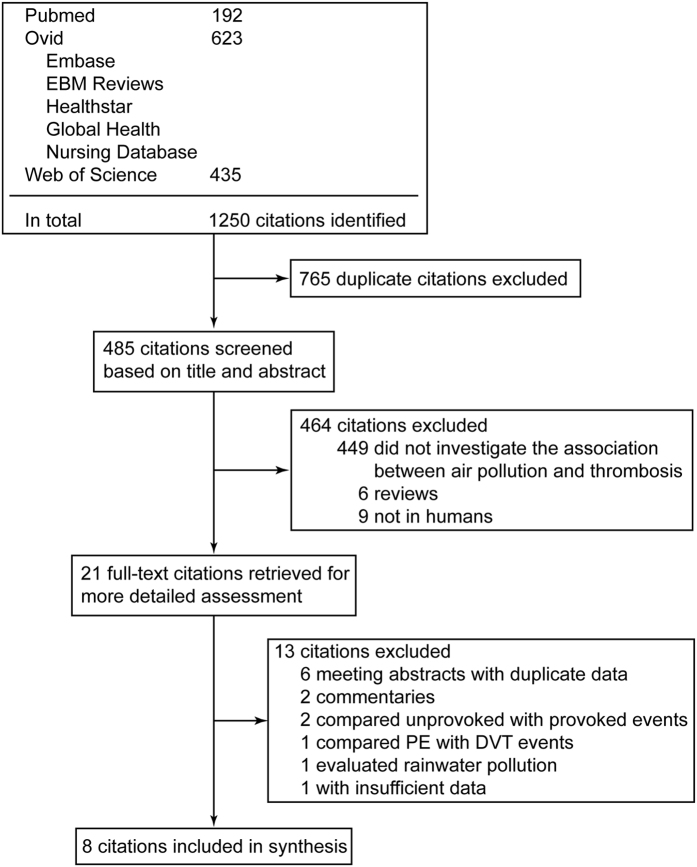
Flowchart of the study selection.

Our initial search yielded 1,250 potential literature citations (Fig. 1). After screening abstracts and in-depth review, 21 citations were selected for further evaluation. Of these, additional data were requested from the authors of 3 citations but obtained for only 2. The remaining one citation with insufficient data was excluded. Altogether 8 citations fulfilled the inclusion criteria^25^,^26^,^27^,^28^,^29^,^30^,^31^,^32^. Interrater agreement for study selection was high (κ = 0.93). The 8 eligible studies included 2 time-series studies, 3 case-crossover studies, 2 prospective cohort studies, and 1 case-control study. Although different methods and study designs were employed, it is reasonable to analyze these studies together because there is a common exposure (air pollution)^33^.

The characteristics of included studies are shown in Table 1. The number of patients or events per study ranged between 302 and 605,242. The study population was predominantly the general population, with the exception of one study that focused on post-menopausal women^28^. Because only two studies (Milojevic 2014 and Dales 2010) investigated the association between CO/SO_2_/O_3_ and venous thrombosis^26^,^30^, these pollutants were excluded from further analysis. One study evaluated both short-term and long-term effects of air pollution^28^, while one study assessed deep vein thrombosis and pulmonary embolism, respectively^30^. Thus, data from the two studies were extracted separately for subgroup analyses.

### Relationship of air pollution and venous thrombosis

**Figure 2:**
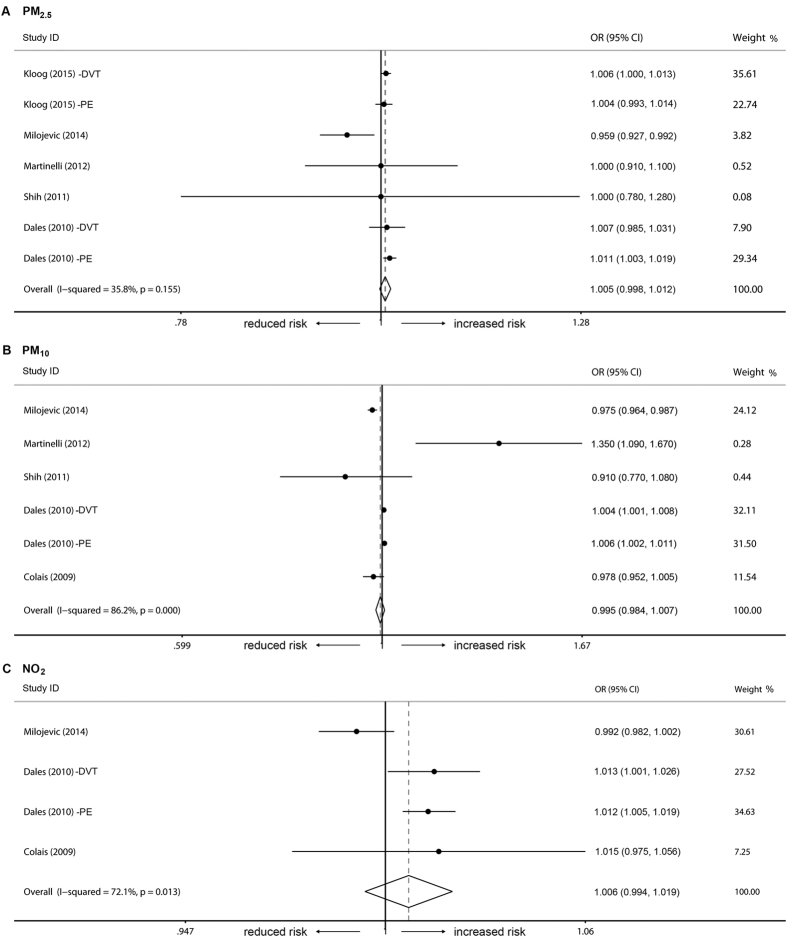
Short-term effects of PM_10_, PM_2.5_, and NO_2_ on venous thrombosis. Individual studies are identified by the author’s last name and year of publication. Venous thrombosis risk was pooled for each increment of 10 μg/m^3^ in pollutant concentration. The size of the ORs data markers is relative to each study weight.

**Figure 3:**
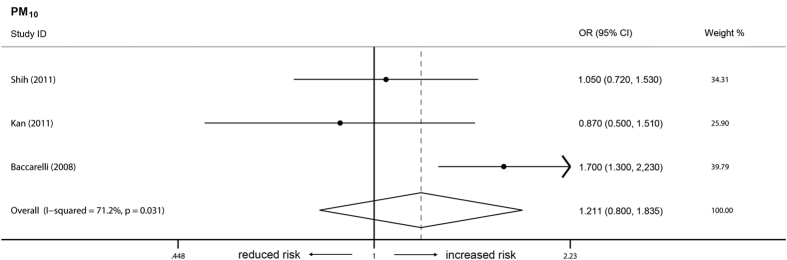
Long-term effects of PM_10_ on venous thrombosis. Individual studies are identified by the author’s last name and year of publication. Venous thrombosis risk was pooled for each increment of 10 μg/m^3^ in pollutant concentration. The size of the ORs data markers is relative to each study weight.

**Figure 4:**
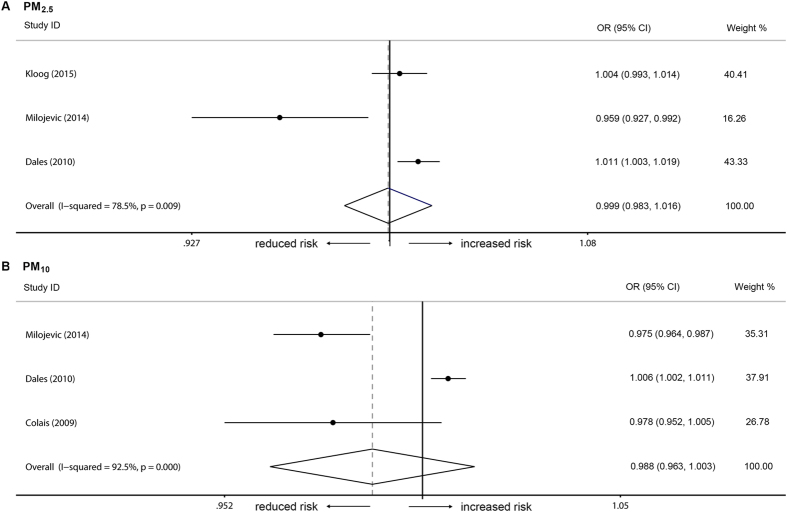
Subgroup analyses on pulmonary embolism. Individual studies are identified by the author’s last name and year of publication. Venous thrombosis risk was pooled for each increment of 10 μg/m^3^ in pollutant concentration. The size of the ORs data markers is relative to each study weight.

In the short-term model, there was no association between the occurrence of venous thrombosis and the three types of air pollutants (Fig. 2A–C). For each 10 μg/m^3^ increment in pollutant concentration, the combined ORs were 0.995 (95% CI = 0.984–1.007, *I*^*2*^ = 86.2%, Q = 9.35, P = 0.155), 1.005 (95% CI = 0.998–1.012, *I*^*2*^ = 35.8%, Q = 36.30, P < 0.001), and 1.006 (95% CI = 0.994–1.019, *I*^*2*^ = 72.1%, Q = 10.74, P = 0.013), respectively, for PM_10_, PM_2.5_, and NO_2_. Based on exposure period and thrombosis location (pulmonary embolism), additional subgroup analyses were performed. Likewise, long-term exposure to the analyzed air pollutant (PM_10_) did not contribute to the occurrence of venous thrombosis (Fig. 3). Additionally there was no association between pulmonary embolism and PM_10_ or PM_2.5_ (Fig. 4A,B). Publication bias (P >0.05 in Egger’s tests and Begg’s tests) was not observed in all these analyzes.

## Discussion

This meta-analysis is the first to assess the association between exposure to major air pollutants and venous thrombosis risk. According to the overall analysis and the subgroup analysis, no significant association was observed between the three air pollutants and venous thrombosis. These results are really unexpected, since a considerable number of experimental studies conducted in animals and in humans have suggested that air pollution may increase the susceptibility to venous thrombosis^34^.

Animal studies demonstrated that exposure to particulate air pollutants could lead to the activation of platelets, the increase in hemostasis factors, histamine release, and the heightened thrombus formation^35^,^36^,^37^. Experiments in humans showed a similar phenomenon. Elevated plasma viscosity was associated with acute changes in PM concentrations, as observed in 3256 randomly selected participants^38^. Healthy adults exposed to traffic-related pollutants had increased plasma levels of homocysteine, an established risk factor for arterial and venous thrombosis^39^. Ambient PM_10_ levels have also been related to platelet aggregation among healthy persons, even 1 to 4 days after exposure^40^. Studies conducted in healthy persons as well as in individuals with venous thrombosis found that prothrombin time was shortened in relation to high levels of ambient PM_10_^32^,^41^.

Nevertheless, our work was consistent with evidence from another recent animal study^42^. Using a murine model, the experimental study showed that acute exposure to PM triggered primary hemostasis activation without substantial secondary hemostasis activation, resulted in arterial but not venous thrombosis. Potential explanations for the discrepancy among the reported studies are described as follows. First, the protocol details greatly vary in regard to the exposure duration, pollutants concentrations, PM compositions, animal models, and preexisting susceptibility. Second, the procoagulant and thrombotic effects of air pollutants were relatively mild, even at concentrations that were much higher than those of the real daily air pollutants. The procoagulant effects observed in experimental studies were too weak to give rise to a pathological venous thrombosis in healthy human beings.

Potential limitations of this study should be considered. First, compared with the investigations on the associations between air pollutants and cardiovascular diseases, the number of available studies in this meta-analysis is small (8 citations). This number is relatively small even though all the major databases were searched with no other restrictions. However, the combined 95% CIs were narrow, which was attributed to the large absolute number of participants and events. Moreover, all of the included studies were of high quality (Table S1). For example, all these studies used multi-pollutant models. Therefore, the findings in this work should be solid. Second, most of the included studies were primarily focused on particulate matters. It is not clear whether other types of air pollution (CO, SO_2_, and O_3_) will have adverse effects on venous thrombosis. Third, to date, the studies reported in literature and those considered in the meta-analysis, are characterized by intrinsic bias. This intrinsic bias includes “exposure bias” due to the difficulty to estimate the personal exposure of each subject enrolled^43^, and “population bias” due to the limited information regarding some peculiar aspects of the study population^44^. Fourth, our conclusion was different from a recently published paper^45^, in which a relationship between air pollution and venous thrombosis was considered. However, in that study, only a systematic review, but no pooled analysis was conducted. In our study, the pooled analyses showed no association in various subgroups (different pollutants, long term or short term effects, for PE only). In addition, we excluded one study^46^ without enough data and another study comparing unprovoked PE with provoked PE^44^. The two excluded studies were of case-control design, with a sample size of only 2,613 or 105. Even if these two studies were included, we believed that they would not change our conclusion, because they would have a very low weight in the pooled analysis.

It is worth noting that all of the available studies were conducted in Western countries, where the median PM_2.5_ concentration is less than 20 μg/m^3^. In developing countries, however, the PM_2·5_ concentrations in urban cities are likely to be up to 100–200 μg/m^3^, such as Beijing, Wuhan, and most of the other large cities in Asia (Figure S1). Will such a high level of air pollution have a marked effect on human blood coagulation and result in venous thrombosis tendency? Thus, relevant studies from these countries, including China, are warranted in the future.

## Conclusions

In conclusion, despite being an important adverse factor for cardiovascular diseases, air pollution may not be a risk factor for venous thrombosis in the general population. More epidemiologic studies are urgently needed to establish the association between air pollution and venous thrombosis in middle-income and low-income countries.

## Additional Information

**How to cite this article**: Tang, L. *et al*. Air pollution and venous thrombosis: a meta-analysis. *Sci. Rep.* **6**, 32794; doi: 10.1038/srep32794 (2016).

## Supplementary Material

Supplementary Information

## Figures and Tables

**Table 1:**
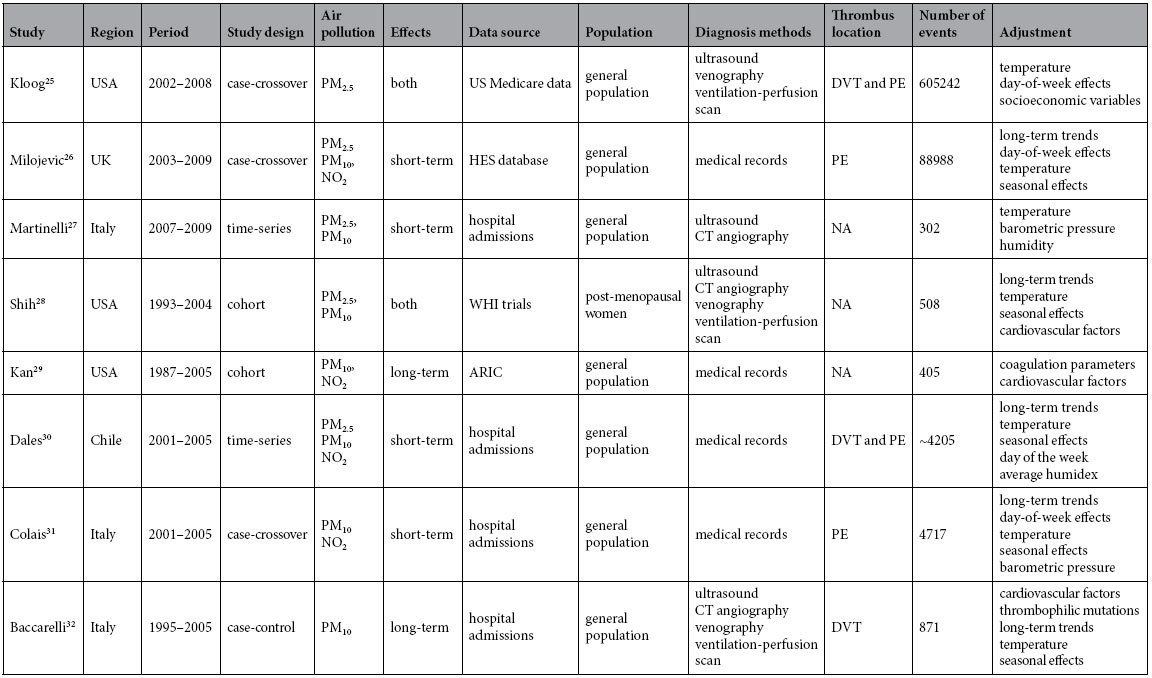
Contextual details of studies included in the meta-analysis.

In the study by Dales 2010, number of events were estimated from mean daily values (1.549 and 0.754) and the study period (2001–2005). Both short-term and long-term effects of air pollution were analyzed by Shih (2011). HES, Hospital Episode Statistics in England and Wales; WHI, the Women’s Health Initiative (WHI) Hormone Therapy trials; ARIC, the Atherosclerosis Risk in Communities study; DVT, deep vein thrombosis; PE, pulmonary embolism; NA, not available.
