# Supplementary material for: Air pollution and venous thrombosis: a meta-analysis
Source: Sci Rep. 2016 Sep 7;6:32794. doi: 10.1038/srep32794 (PMC5013712; doi:10.1038/srep32794)
Supplement: Supplementary Information [file srep32794-s1.pdf]

Supplemental material

Air pollution and venous thrombosis: a meta-analysis

Liang Tang,[1,2*] Qing-Yun Wang,[1*] Zhi-Peng Cheng,[1] Bei Hu,[1,2] Jing-Di Liu,[1] and Yu Hu[1,2]

[1]Institute of Hematology, Union Hospital, Tongji Medical College, Huazhong University of Science and Technology, Wuhan, Hubei, 430022, China;

[2]Collaborative Innovation Center of Hematology, Union Hospital, Huazhong University of Science and Technology, Wuhan, 430022, China;

[*]The first two authors contributed equally to this work

Address correspondence to: Yu Hu; Tel.: +86 27 85726335; fax: +86 27 85726387. E-mail: dr_huyu@126.com

Search Criteria

Ⅰ **Pubmed**

((((((((((((((((((air pollution) OR air pollution[MeSH Terms]) OR particulate matter) OR particulate matter[MeSH Terms]) OR nitrogen dioxide) OR nitrogen dioxide[MeSH Terms]) OR carbon monoxide) OR carbon monoxide[MeSH Terms]) OR sulphur dioxide) OR sulphur dioxide[MeSH Terms]) OR ozone) OR ozone[MeSH Terms]) OR "pm 2 5") OR "pm2 5") OR "pm 10") OR "pm10" OR haze))) AND ((((((((((Venous thrombosis) OR Venous thrombosis[MeSH Terms]) OR venous thromboembolism) OR venous thromboembolism[MeSH Terms]) OR deep vein thrombosis) OR deep vein thrombosis[MeSH Terms]) OR pulmonary embolism) OR pulmonary embolism[MeSH Terms]) OR pulmonary thromboembolism) OR pulmonary thromboembolism[MeSH Terms])

Ⅱ **Ovid**

including

Embase (1947 to present)

EBM Reviews (Cochrane DSR, ACP Journal Club, DARE, CCTR, CMR, HTA, and NHSEED)

Ovid Healthstar (1966 to present)

Global Health (1910 to present)

Ovid Nursing Database (1946 to present)

1 (deep vein thrombosis OR deep venous thrombosis OR Venous Thrombosis OR Vein Thrombosis).ti

2 exp deep vein thrombosis/

3 Vein Thrombosis/

4 pulmonary embolism.ti OR exp lung embolism/

5 venous thromboembolism.ti OR exp Venous Thromboembolism/

6 venous thromboembolic disorder*.ti OR venous thromboembolic disease*.ti OR venous thrombotic.ti

7 or/1-6

8 (Venous thrombosis venous thromboembolism deep vein thrombosis pulmonary embolism pulmonary thromboembolism).af.

9 7 or 8

10 Exp air pollution.mp. or Air Pollution/

11 Exp particulate matter.mp. or Particulate Matter/

12 Exp nitrogen dioxide.mp. or Nitrogen Dioxide/

13 Exp carbon monoxide.mp. or Carbon Monoxide/

14 Exp sulphur dioxide.mp. or Sulphur Dioxide/

15 Exp ozone.mp. or Ozone/

16 Exp PM 2.5 or PM2.5 or PM 10 or PM10

17 Exp haze

18 or/10-17

19 9 and 18

Ⅲ Web Of Science

"air pollution" OR "particulate matter" OR "nitrogen dioxide" OR "carbon monoxide" OR "sulphur dioxide" OR "ozone" OR "pm 2 5" OR "pm2 5" OR "pm 10" OR "pm10" OR "haze" AND "Venous thrombosis" OR "venous thromboembolism" OR "deep vein thrombosis" OR "pulmonary embolism" OR "pulmonary thromboembolism"

Figure S1. PM concentrations in China. (A) Monthly mean levels of PM10 in 4 large cities in China in 2014. (B) Monthly mean levels of PM2.5 in 4 large cities in China in 2014. Data were obtained and re-calculated from the China Air Quality Monitoring Platform (http://aqistudy.sinaapp.com/). Black line, Beijing (in North); blue line, Wuhan (in South); red line, Shanghai (in East); green line, Chongqing (in West).

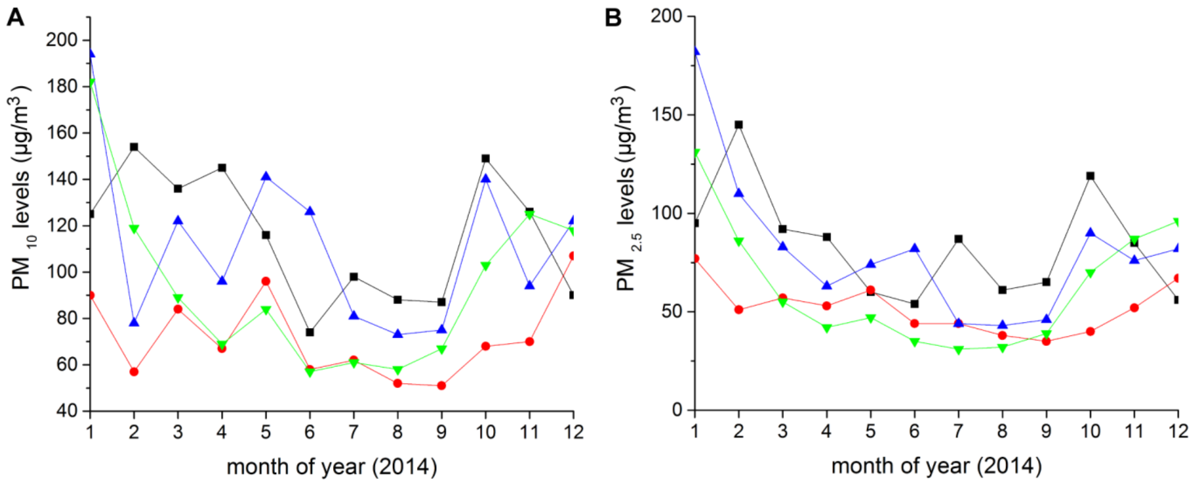

Table S1. Summary of study quality.

Study	VT diagnosis	Pollutant measure	Study area	Total population	Multiple lags	Repeated events	Temperature	Time trends	Season	Other factors	Quality (scores)
Kloog 2015	1	1	1	1	1	0	1	1	1	1	9
Milojevic 2014	1	1	1	1	1	0	1	1	1	1	9
Martinelli 2012	1	1	0	1	1	0	1	1	1	1	8
Shih 2011	1	1	1	0	1	1	1	1	1	1	9
Kan 2011	1	1	1	1	1	1	1	1	1	1	10
Dales 2010	1	1	1	1	1	0	1	1	1	1	9
Colais 2009	1	1	1	1	1	0	1	1	1	1	9
Baccarelli 2008	1	1	0	1	1	1	1	1	1	1	8

Multiple lags defined as studies evaluated pollutant levels in a distributed lag model beyond lag of 1 day (short-term exposure) or lag of 1 year (long-term exposure). Complete evaluation of study area defined as a study where the unit of analysis for the exposure matched that of the outcome. Repeated events defined as multiple hospitalizations were controlled in the analysis. VT, venous thrombosis.
